# Oleic Acid and Palmitic Acid from *Bacteroides thetaiotaomicron* and *Lactobacillus johnsonii* Exhibit Anti-Inflammatory and Antifungal Properties

**DOI:** 10.3390/microorganisms10091803

**Published:** 2022-09-08

**Authors:** Rogatien Charlet, Chrystelle Le Danvic, Boualem Sendid, Patricia Nagnan-Le Meillour, Samir Jawhara

**Affiliations:** 1 UMR 8576—UGSF—Unité de Glycobiologie Structurale et Fonctionnelle, Centre National de la Recherche Scientifique, F-59000 Lille, France; 2 Institut National de la Santé et de la Recherche Médicale U1285, University of Lille, F-59000 Lille, France; 3 Medicine Faculty, University of Lille, F-59000 Lille, France; 4 Eliance, R&D, 149 rue de Bercy, F-75012 Paris, France

**Keywords:** colitis, fatty acids, oleic acid, palmitic acid, inflammatory response, dextran sulfate sodium, *Candida glabrata*, *Bacteroides thetaiotaomicron*, *Lactobacillus johnsonii*, fungal overgrowth

## Abstract

A decrease in populations of *Bacteroides thetaiotaomicron* and *Lactobacillus johnsonii* is observed during the development of colitis and fungal overgrowth, while restoration of these populations reduces inflammatory parameters and fungal overgrowth in mice. This study investigated the effect of two fatty acids from *B. thetaiotaomicron* and *L. johnsonii* on macrophages and Caco-2 cells, as well as their impact on the inflammatory immune response and on *Candida glabrata* overgrowth in a murine model of dextran sulfate sodium (DSS)-induced colitis. Oleic acid (OA) and palmitic acid (PA) from *L. johnsonii* and *B. thetaiotaomicron* were detected during their interaction with epithelial cells from colon samples. OA alone or OA combined with PA (FAs) reduced the expression of proinflammatory mediators in intestinal epithelial Caco-2 cells challenged with DSS. OA alone or FAs increased FFAR1, FFAR2, AMPK, and IL-10 expression in macrophages. Additionally, OA alone or FAs decreased COX-2, TNFα, IL-6, and IL-12 expression in LPS-stimulated macrophages. In the DSS murine model, oral administration of FAs reduced inflammatory parameters, decreased *Escherichia coli* and *Enterococcus faecalis* populations, and eliminated *C. glabrata* from the gut. Overall, these findings provide evidence that OA combined with PA exhibits anti-inflammatory and antifungal properties.

## 1. Introduction

Inflammatory bowel disease (IBD), which includes Crohn’s disease (CD) and ulcerative colitis, is a chronic disorder resulting from deregulation of the immune response to the gut microbiota in genetically susceptible individuals [1,2]. Alteration of the intestinal microbiota, including *Candida* spp., plays an important role in IBD [3]. *Candida albicans* and *Candida glabrata* are opportunistic fungal pathogens that can cause invasive fungal infections [4,5]. Systemic *C. glabrata* infections are associated with higher mortality than *C. albicans* infections [5]. Anti-fungal-glycan antibodies, known as ASCA (anti-*Saccharomyces cerevisiae* mannan antibodies), ALCA (anti-laminaribioside carbohydrate antibodies), and ACCA (anti-chitobioside carbohydrate antibodies), were recognized as serological markers of CD, but subsequent studies established that they can also be generated during *Candida* infection, suggesting a link between CD gut dysbiosis and endogenous opportunistic fungal species [6,7]. In addition, experimental studies have shown that intestinal inflammation increases *C. albicans* overgrowth, and that *C. albicans* in turn accentuates inflammation in a murine model of DSS-induced colitis [8,9]. It has been reported that intestinal inflammation and fungal overgrowth promote a change in the biodiversity of the gut microbiota, including *Bacteroides thetaiotaomicron* and *Lactobacillus johnsonii* populations. These were both highly affected during the development of colitis and overgrowth of *C. glabrata* [10]. Restoration of these two anaerobic bacteria attenuated the development of colitis and *C. glabrata* growth in mice with DSS-induced colitis [11].

Changes in microbial metabolites have been implicated in IBD, such as short-chain fatty acids (SCFAs), which are produced by microbial fermentation of dietary fiber and are significantly reduced in IBD [12,13]. Stool samples from IBD patients typically had less acetate, butyrate, and propionate, while lactate and pyruvate were increased [12]. Further evidence of imbalanced host–microbe interactions in IBD are dietary and environmental factors that are implicated in the perpetuation of IBD [14]. A diet high in processed foods and fatty foods has been implicated in the recent increase in incidence of IBD, particularly in the Western world [15]. A mouse model mimicking a Western-style diet high in fat and sugar revealed the overgrowth of proinflammatory Proteobacteria such as *Escherichia coli*, a decrease in protective bacteria, and a significantly reduction in SCFA concentrations [16]. Furthermore, colonocyte metabolism of SCFA, in particular butyrate, was impaired in DSS-induced colitis, while in vitro supplementation of the microbiota of CD patients with butyrate-producing bacteria resulted in higher butyrate production, supporting an improvement in the integrity of the intestinal epithelial barrier [17,18]. SCFA and medium-chain fatty acids (FAs) displayed not only anti-inflammatory properties, but also antimicrobial activities, including against *Candida* spp. [19,20].

This study investigated the effects of two FAs (oleic acid and palmitic acid) detected from *B. thetaiotaomicron* and *L. johnsonii* on intestinal epithelial Caco-2 cells challenged with DSS and on macrophages. Additionally, the impact of these two FAs on *Candida* spp. was also investigated in terms of viability, adhesion, fungal growth, and biofilm formation, and on modulation of the inflammatory immune response in a murine model of DSS-induced colitis.

## 2. Materials and Methods

### 2.1. Fungal and Bacterial Cultures

The fungal strains used were *C. glabrata* wild-type (ATCC; *Cg*), *C. albicans* SC5314, and *C. albicans* bioluminescent strain [21,22]. These yeasts were grown in YPD medium (1% yeast extract, 1% peptone, 1% dextrose) on a rotary shaker for 18 h at 37 °C [23]. The fungal culture obtained was then centrifuged at 2500 rpm for 5 min and washed twice in phosphate-buffered saline (PBS; Gibco; 14200-067). Bacterial strains of *B. thetaiotaomicron* and *L. johnsonii* were isolated from mouse stools on *Bacteroides* bile esculin agar and De Man, Rogosa, and Sharpe culture medium at 37 °C for 48 h under anaerobic conditions (Anaeroben^TM^ 3.5L; ref: AN0035A; Thermo Scientific, Waltham, MA, USA). The bacteria isolated were identified using MALDI-TOF (Microflex-Bruker Daltonics, Billerica, MA, USA) [11].

### 2.2. Ex Vivo Detection of Bacterial Metabolites Using Gas Chromatography–Mass Spectrometry (GC-MS)

The colons were removed from C57BL/6 mice and washed with PBS followed by RPMI supplemented with 10% fetal calf serum and 1% antibiotics (penicillin G + streptomycin, 0.1 mg/mL) [24]. Coincubation of these two bacteria was then carried out for 12 h under different conditions: colon alone; colon + *Cg* (as a control); colon + *B. thetaiotaomicron* + *L. johnsonii* (BtLj; 10^7^ cells); and colon + BtLj + *Cg* (10^7^ cells). After incubation, the colons were collected, and 1 mL of each supernatant was extracted with CHCl_3_/MeOH (2:1). The extract was then centrifuged at 1500 rpm for 10 min at 4 °C, and the chloroform phase was recovered and evaporated in a Speedvac to concentrate the sample and obtain the FAs. To facilitate their identification, FAs were derivatized by transesterification to obtain the fatty acid methyl esterified (FAME). Chloroform (100 µL) was added to the tube containing the dry sample, then 1.5 mL of MeOH/HCl (0.5 N) was added, followed by a dry bath at 80 °C for 18 h. Once the tubes had cooled, three successive extractions were performed using heptane before analysis using GC-MS (ThermoFisher Scientific). Following this analysis, the two FAs were identified using GC-MS (oleic acid (OA) and palmitic acid (PA)).

### 2.3. Expression of Proinflammatory Cytokines and Innate Immune Receptors by Macrophages

THP-1 cells were differentiated into macrophages by adding phorbol-12-myristate13-acetate (PMA: Sigma-Aldrich, Saint-Quentin-Fallavier, France) at a concentration of 200 ng/mL for 72 h. The macrophages were then plated at a concentration of 10^6^ cells/well for 24 h in RPMI medium [23]. Lipopolysaccharide (LPS) was then added to the macrophages at a concentration of 250 ng/mL (LPS from *E. coli* O111: B4; Sigma-Aldrich, France) for 6 h. In parallel, OA (TCI; Tokyo, Japan) and PA (TCI; Japan) were added at a concentration of 25 µg/mL. Macrophages alone were used as a control. Subsequently, macrophages were harvested in RA1 buffer to perform mRNA extraction followed by RT-PCR and q-PCR. For quantitative PCR reactions, cDNA products were amplified using SYBR green real-time PCR master mix reagent. The SYBR green dye intensity was determined using one-step software. The used primers are provided in Table S1 in the Supplementary Materials. The mRNA levels were normalized to the reference gene (GAPDH (mRNA)) and are reported as fold-change in expression over the control (macrophages alone). For the measurement of proinflammatory cytokines using ELISA, 10^6^ THP-1 cells were differentiated into macrophages by adding PMA at a concentration of 200 ng/mL for 72 h. The macrophages were then incubated for 24 h in RPMI medium. LPS was then added to the macrophages at a concentration of 250 ng/mL for 16 h, and FAs were added at a concentration of 25 μg/mL. The supernatants were collected from each well. The concentrations of TNFα (InvitrogenTM NovexTM Human TNF alpha ELISA Kit), IL-6 (ELISA MAXTM Deluxe Set Human IL-6; 430504; BioLegend, San Diego, CA, USA), and IL-1β (ELISA MAXTM Deluxe Set Human IL-1β; 437004; BioLegend) in the supernatants were measured using ELISA according to the manufacturer’s instructions. The concentrations of TNFα, IL-6, and IL-1β were then determined based on standard curves provided in the ELISA kits, and the results were expressed as “Fold Release” by normalizing with the positive control (macrophages + LPS).

### 2.4. Migration of Macrophages through Human Intestinal Epithelial Caco-2 Cells Treated with DSS and Fatty Acids

Caco-2 cells (10^6^ cells) were added to inserts (HTS Transwell 96-well; Corning; 3387) in the presence of Dulbecco’s Modified Eagle Medium (DMEM) for 24 h [25]. Dextran sulfate sodium (DSS; 1.5%; MP Biomedicals, LLC, Eschwege, Germany) was then added to each well for 24 h. Caco-2 cells were then treated with FA at a concentration of 25 µg/mL. In parallel, THP-1 cells were differentiated into macrophages using PMA at a concentration of 200 ng/mL for 72 h. After different washings with PBS, the macrophages were maintained in RPMI for 24 h. They were then labeled with fluorescent calcein (Invitrogen; Villebon sur Yvette, France) and added to each insert at a concentration of 10^5^ macrophages/insert. RPMI (150 µL) containing 10^5^ *C. albicans* yeast cells was then added to the lower chamber, and the plates were incubated at 37 °C in 5% CO_2_ overnight. After overnight migration, the upper chamber of the Transwell inserts containing nonmigrated macrophages was removed from the plate, and migrated macrophages present on *C. albicans* cells were determined by measuring fluorescence using a fluorometer (FLUOstar^®^ Omega; BMG Labtech, Saitama, Japan). Macrophage migration through Caco-2 cells untreated with DSS was assigned a value of 100%, corresponding to a healthy intestinal barrier, while the value 200% corresponded to a destroyed intestinal barrier.

### 2.5. Adhesion of C. glabrata to Human Intestinal Epithelial Caco-2 Cells Treated with Fatty Acids

Caco-2 cells (150 µL containing 5 × 10^5^ cells) were added to each well of a 96-well plate (Greiner Bio-One, Kremsmünster, Austria). The plate was incubated at 37 °C in a humidified atmosphere containing 5% CO_2_. Once the Caco-2 cells were confluent, the intestinal cells were treated with FA at a concentration of 25 μg/mL. *C. glabrata* yeast cells (100 µL containing 10^5^ cells) labeled with calcein (Invitrogen; France) were added to each well. After 2 h of incubation and different washings with PBS, fluorescence was measured using a fluorometer (FLUOstar Omega; BMG Labtech).

### 2.6. Effect of Fatty Acids on Candida *spp.*

The *C. albicans* bioluminescent strain (100 µL containing 10^6^ cells) was added to each well of a 96-well plate (Greiner Bio-One) [26]. OA and PA were added at various concentrations (250, 100, 50, 25, and 10 µg/mL). Finally, coelenterazine was added at a concentration of 2 µM (Coelenterazine, ABP Biosciences/tebu-bio, Le Perray-en-Yvelines, France). After 0, 1, and 2 h incubation, bioluminescence was measured using a fluorometer (FLUOstar Omega; BMG Labtech). *C. albicans* viability was considered to be 100% when the fungal cells were unchallenged with any FA treatment (100% corresponding to a control group receiving PBS only). We then normalized all of the results obtained under the various conditions according to this control. To test the viability of *C. glabrata* in the presence of FA, 1 mL RPMI containing 10^5^ *C. glabrata* cells in the presence or absence of FA (250 or 25 µg/mL) was used. Kinetics were carried out after coincubation for different times (0, 1, and 2 h) at 37 °C. Different dilutions were then performed in order to obtain a volume of 100 µL per Sabouraud agar Petri dish. The number of fungal colonies was determined in each Petri dish after 24 h of incubation at 37 °C. In parallel, caspofungin (50 mg; OHRE Pharma, Tours, France) at a concentration of 100 µg/mL was used as a control.

### 2.7. Effect of Fatty Acids on Fungal Biofilm Formation

RPMI (200 µL) containing 10^7^ *C. albicans* SC5314 or *C. glabrata* ATCC 2001 cells was added to each well of a 96-well plate (Greiner Bio-One; 655101). After 1.5 h of incubation at 37 °C, different washings with PBS were performed. RPMI (200 µL) containing the FAs was added to each well. The biofilm was then measured after 0, 4, 8, and 24 h of incubation. The plate was washed with PBS and left to dry for 2 h at 37 °C before adding 110 µL of crystal violet (0.4%; Fluka, Gillingham, UK; 61135) for 45 min. The plate was then washed four times with 300 µL water before adding 200 µL of ethanol. Absorbance of the decolorization solution was measured at 550 nm using a spectrophotometer (FLUOstar; BMG Labtech, Champigny sur Marne, France). Caspofungin at a concentration of 100 μg/mL was used as a control. The biofilm analyzed at T0 represented the basal rate for each condition and corresponded to a rate of 100%. Thus, each condition assessed at T4, T8, and T24 was normalized according to that of the biofilm observed at T0.

### 2.8. Animal Model

Female C57BL/6 mice aged 3–4 months and certified free from infection were used (Janvier Laboratories, Le Genest-Saint-Isle, France). The mice were kept at 21 °C with free access to water and food and a 12/12 h light/dark cycle in the animal facility of the Faculty of Medicine, Lille University. We used hermetically sealed and filtered single-use plastic cages that were only opened in rooms containing no other animals and were disinfected between each series of experiments. All experiments were performed in accordance with the decrees relating to the ethics of animal experimentation (Decree 86/609/EC), as well as according to Protocol 00550.05.

On day 0, a single oral gavage of 300 μL PBS containing 5 × 10^7^ cells of *C. glabrata* was given to each mouse. The animals received an oral gavage of 250 μL PBS containing the FA (OA and PA) at a concentration of 1 mg/kg/mouse during the first 5 days. To induce chemical colitis in the mice, DSS at a concentration of 1.5% was added to water for the entire duration of the experiment. The optimal effect of DSS was reached on the sixth day. The animals were separated into several groups with free access to water containing 1.5% DSS. The control group (CTL) represented mice receiving water. The groups *C. glabrata* (*Cg*) alone or FAs alone represented control groups without DSS treatment. D corresponded to mice receiving DSS to induce chemical colitis, D*Cg* to mice receiving DSS and *C. glabrata*, and D*Cg*FA to mice treated with FA and challenged with DSS and *C. glabrata*.

During the experiments, the animals were weighed, and stool samples were collected every 2 days. The stool samples were weighed, homogenized in 1 mL PBS, and then serially diluted in 1 mL 1X PBS [22,27]. A volume of 100 µL was then added to Petri dishes containing Sabouraud agar. A bacterial culture was performed on MacConkey and bile esculin azide medium for Enterobacteriaceae and Enterococci, respectively. After 24 h of incubation at 37 °C fungal growth was recorded as number of colony-forming units (CFU)/mg of stool. To identify bacterial colonies in the Petri dishes, 1.5 µL of matrix solution (α-cyano-4-hydroxycinnamic acid; Bruker Daltonics) dissolved in 50% acetonitrile, 47.5% water, and 2.5% trifluoroacetic acid was added to the bacterial colonies, and they were then analyzed using mass spectrometry (MALDI-TOF; Microflex-Bruker Daltonics). On day 14, the mice were sacrificed by cervical dislocation and the colon, stomach, and caecum were collected to determine the fungal load. After different washings with PBS, the colon, stomach, and caecum were weighed and homogenized in 1 mL PBS (TURRAX, T10 basic ULTRA-TURRAX^®^, IKA^®^). Each sample (100 µL) was plated on Sabouraud agar for 24 h at 37 °C. The result was noted as number of CFU/mg of the organ.

### 2.9. Clinical and Histologic Scores for Inflammation

Clinical scores were assessed independently by two investigators blinded to the study protocol, as described previously [28,29]. Four scores (behavior, body weight, stool consistency, and bleeding) were added together, resulting in a total clinical score ranging from 0 (healthy) to 6 (maximum colitis activity). For analysis of the histologic score, the colon sample from each animal was fixed overnight in 4% paraformaldehyde-acid at 4 °C. The colon samples were then embedded in paraffin at 60 °C overnight. The samples were fixed, cut in a microtome (4 µm thick; RM2245, Leica, Wetzlar, Germany), and scanned with an Axio-Scan.Z1 (Zeiss, Oberkochen, Germany). For the histologic score, 0 = no epithelial lesion and no leukocyte infiltrates; 3 = presence of epithelial lesion, edema, or leukocyte infiltrates; 6 = presence of epithelial lesions, edema, and leukocyte infiltrates.

### 2.10. Real-Time mRNA Quantification of Proinflammatory Cytokines and Innate Immune Receptors

RNA was extracted from the colon samples using the NucleoSpin RNA^®^ protocol (Macherey-Nagel, Düren, Germany). For each sample, mRNA (in µg/µL) was measured with a spectrophotometer (Nanodrop 1000; Thermo Scientific). The ratio of each of our samples was >2.0, which was a sign of pure RNA. The cDNA synthesis was performed using a high-capacity DNAc reverse-transcription (RT) master mix (Applied Biosystems, Waltham, MA, USA). For a final volume of 10 µL/sample, the mix contained 2 µL 10X RT buffer, 2 µL 10X RT random primers, 0.8 µL 25X dNTP mix, 1 µL reverse transcriptase, and 4.2 µL RNase-free H_2_O. PCR was performed in a 96-well microplate (MicroAmp Fast Optical 96-well; Applied Biosystems). For a final volume of 12 µL/well of each sample, a mix was made containing 0.25 µL of forward and reverse primer, 6 µL of SYBR green (Applied Biosystems), 3 µL of RNase-free H_2_O, and 2.5 µL of cDNA from the samples. SYBR green dye intensity was determined using one-step software. The used primers are provided in Table S2 in the Supplementary Materials. The mRNA levels were normalized to the reference gene (POLR2A (mRNA)) and are reported as fold-change in expression over the control group (CTL).

### 2.11. Statistical Analysis

The data are presented as the mean ± standard deviation (SD) of the individual experimental groups. Statistical comparisons were performed using the nonparametric Mann–Whitney test or ANOVA,

All statistical analyses were performed using GraphPad Prism^®^ 7.0 software (GraphPad software, San Diego, CA, USA). The *p*-values < 0.05, 0.01, or 0.001 were considered statistically significant.

## 3. Results

### 3.1. Characterization and Identification of Two Fatty Acids from B. thetaiotaomicron and L. johnsonii by GC-MS

**Figure 1:**
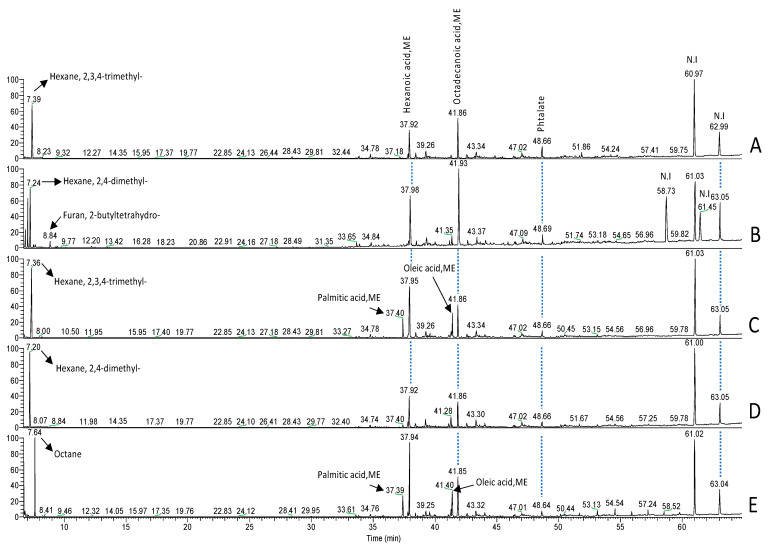
Representative data of the analysis obtained using GC-MS. (**A**) Analysis of RPMI medium. (**B**) Analysis of a control mouse colon sample. (**C**) Analysis of a mouse colon sample incubated with *L. johnsonii* and *B. thetaiotaomicron*. (**D**) Analysis of a mouse colon sample incubated with *C. glabrata*. (**E**) Analysis of a mouse colon sample incubated with *L. johnsonii, B. thetaiotaomicron,* and *C. glabrata*. The retention time (RT) peak of 6.85 min corresponded to tetrahydrofuran, 2-methyl-5-methyl; while the RT peak of 7.04 min was not identified in chromatogram B.

Two FAs were identified from *B. thetaiotaomicron* and *L. johnsonii* during their interaction with epithelial cells from mouse colons (Figure 1). In contrast to the controls (RPMI, colon alone, or colon incubated with *C. glabrata*), two FAs were detected using GC-MS in colon extract incubated with these two anaerobic bacteria. The first FA was detected at a peak of 37.4 min, corresponding to palmitic acid methyl ester (PAME); while the second was observed at 41.4 min, corresponding to oleic acid methyl ester (OAME). This GC-MS analysis indicated the presence of PA and OA following bacterial interaction with the colon samples.

### 3.2. Effect of These Two Fatty Acids on the Modulation of Proinflammatory Cytokine Expression in Caco-2 Cells Challenged with DSS and on the Migration of Macrophages through DSS-Challenged Caco-2 Cells

**Figure 2:**
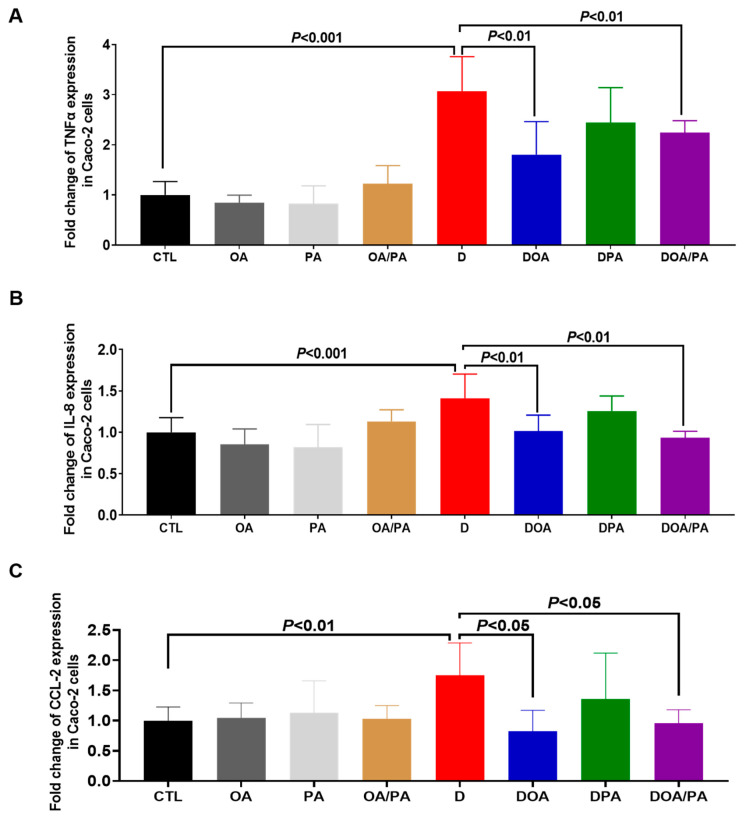
Effect of fatty acids on the modulation of proinflammatory cytokine expression in DSS-treated Caco-2 cells. Relative expression levels of (**A**) TNFα, (**B**) IL-8, and (**C**) CCL-2 mRNA in Caco-2 cells challenged with 1.5% DSS. CTL: control group (Caco-2 cells alone); OA: Caco-2 cells treated with OA; PA: Caco-2 cells treated with PA; OA/PA: Caco-2 cells treated with PA and OA; D: Caco-2 cells challenged with 1.5% DSS; DOA: Caco-2 cells challenged with 1.5% DSS and treated with OA; DPA: Caco-2 cells challenged with 1.5% DSS and treated with PA; DOA/PA: Caco-2 cells challenged with 1.5% DSS and treated with PA and OA. The results were obtained from three independent experiments.

**Figure 3:**
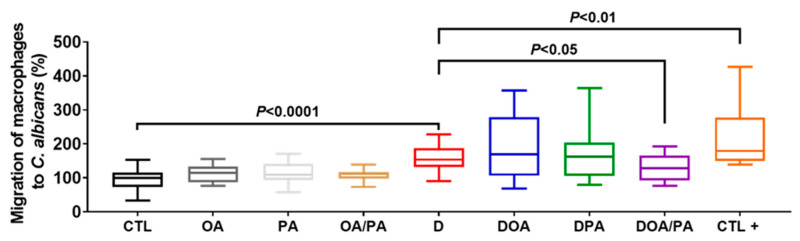
Migration of macrophages to *C. albicans* through Caco2 cells challenged with DSS and treated with fatty acids (FAs). CTL: control (migration of macrophages through Caco-2 cells untreated with DSS); OA: migration of macrophages through Caco-2 cells treated with OA only and unchallenged with DSS; PA: migration of macrophages through Caco-2 cells treated with PA only and unchallenged with DSS; OA/PA: migration of macrophages through Caco-2 cells treated with both FAs and unchallenged with DSS; D: migration of macrophages through Caco-2 cells challenged with DSS; DOA: migration of macrophages through Caco-2 cells challenged with DSS and treated with OA; DPA: migration of macrophages through Caco-2 cells challenged with DSS and treated with PA; DOA/PA: migration of macrophages through Caco-2 cells challenged with DSS and treated with both FAs; CTL+: positive control (migration of macrophages without Caco-2 cells). The results were obtained from three independent experiments.

Caco-2 cells treated with the fatty acids did not show a significant variation in the expression of TNFα, IL-8, and CCL2 when compared to untreated cells (Figure 2). A significant increase in the expression of these cytokines was seen when the intestinal cells were challenged with 1.5% DSS for 24 h (Figure 2). However, Caco-2 cells treated with FAs and challenged with DSS showed a significant decrease in the expression of these proinflammatory mediators when compared to untreated Caco-2 cells challenged with DSS (Figure 2). The migration of macrophages through Caco-2 cells unchallenged with DSS (CTL-) was assigned a value of 100%, while the positive control corresponded to easy migration of macrophages without Caco-2 cells. Macrophage migration through Caco-2 cells with DSS was assigned a value of 200% (D). Treatment of epithelial cells with either OA or PA did not reduce macrophage migration when compared to untreated Caco-2 cells challenged with DSS (Figure 3). Interestingly, treatment of intestinal cells with both FAs significantly reduced the migration of macrophages through Caco-2 cells challenged with DSS, indicating that a combination of these two FAs improved the intestinal epithelial barrier (Figure 3).

### 3.3. Effect of Fatty Acids on the Modulation of Proinflammatory Mediators and Receptors in Macrophages

**Figure 4:**
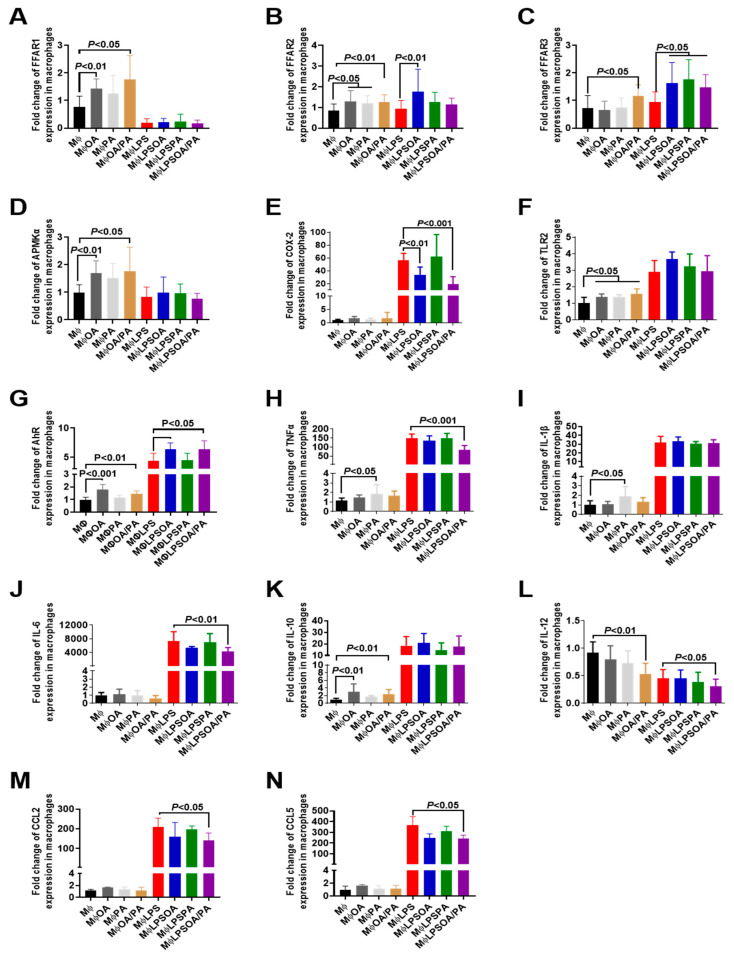
Expression of proinflammatory mediators and receptors in macrophages treated with fatty acids and challenged with lipopolysaccharide (LPS). (**A**–**N**) Relative expression levels of FFAR1, FFAR2, FFAR3, AMPKα, COX-2, TLR2, AhR, TNFα, IL-1β, IL-6, IL-10, IL-12, CCL2, and CCL5 mRNA, respectively, in macrophages. MΦ: control group (macrophages alone); MΦOA, MΦPA, and MΦOA/PA: macrophages treated with OA, PA, or FAs (OA/PA), respectively; MΦLPS: positive control (macrophages exposed to LPS); MΦLPSOA, MΦLPSPA, and MΦLPSOA/PA: macrophages challenged with LPS and treated with OA, PA, or FAs (OA/PA), respectively. The results were obtained from three independent experiments.

Regarding the receptors mediating FAs as signaling molecules, OA or OA+PA (FAs) increased the expression of FFAR1 and FFAR2 in macrophages. The addition of OA or FAs to macrophages significantly increased the expression of 5′ AMP-activated protein kinase (AMPK), TLR2, AhR, and IL-10, while there was no change in proinflammatory cytokines except for IL-12 expression, which was decreased significantly in macrophages treated with FAs (Figure 4). PA increased FFAR2, TLR2, TNFα, and IL-1β expression in macrophages.

In LPS-stimulated macrophages, OA, PA, or FAs increased the expression of FFAR3, whereas the expression of FFAR2 was observed only in the presence of OA. LPS-stimulated macrophages resulted in the expression of TLR2, TLR4, TLR8, AhR, COX-2, MyD88, NF-kB, TNFα, IL-1β, IL-6, IL-8, CCL2, and CCL5 (Figure 4 and Supplementary Materials Figure S1). Treatment of LPS-stimulated macrophages with FAs significantly decreased COX-2, TNFα, IL-6, IL-12, CCL2, and CCL5, while the expression of FFAR1 was not affected in LPS-stimulated macrophages treated with FAs (Figure 4 and Supplementary Materials Figure S1). Additionally, a high expression of AhR was observed in LPS-stimulated macrophages treated with OA or FAs. Protein expression levels of TNFα and IL-6 revealed the anti-inflammatory effect of FAs in macrophages treated with LPS (Supplementary Materials Figure S2).

### 3.4. Role of These Two Fatty Acids in Pathogen–Epithelial Cell Interactions

**Figure 5:**
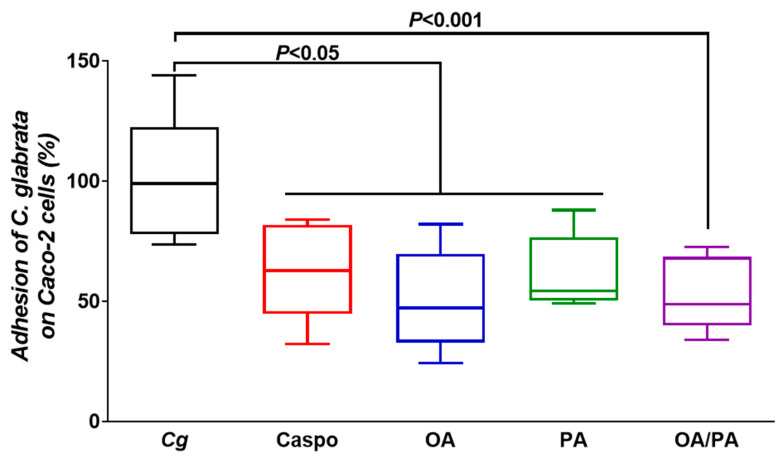
Effect of fatty acids on the adhesion of *C. glabrata* to Caco-2 cells. *Cg*: control (Caco-2 cells challenged with *C. glabrata*); Caspo: standard control (Caco-2 cells challenged with *C. glabrata* and treated with caspofungin); OA, PA, OA/PA: Caco-2 cells challenged with *C. glabrata* and treated with OA, PA, or FAs (OA/PA), respectively. The results were obtained from two independent experiments.

To evaluate whether these two FAs could reduce the adhesion of pathogens, in particular *Candida* spp., to Caco-2 cells, FAs at a concentration of 25 µg/mL were added to Caco-2 cells, and the epithelial cells were then challenged with *C. glabrata*. A decrease in the adhesion of *C. glabrata* to intestinal cells treated with OA, PA, or FAs was observed, indicating that these two FAs protected the intestinal epithelial barrier from pathogen adhesion (Figure 5).

**Figure 6:**
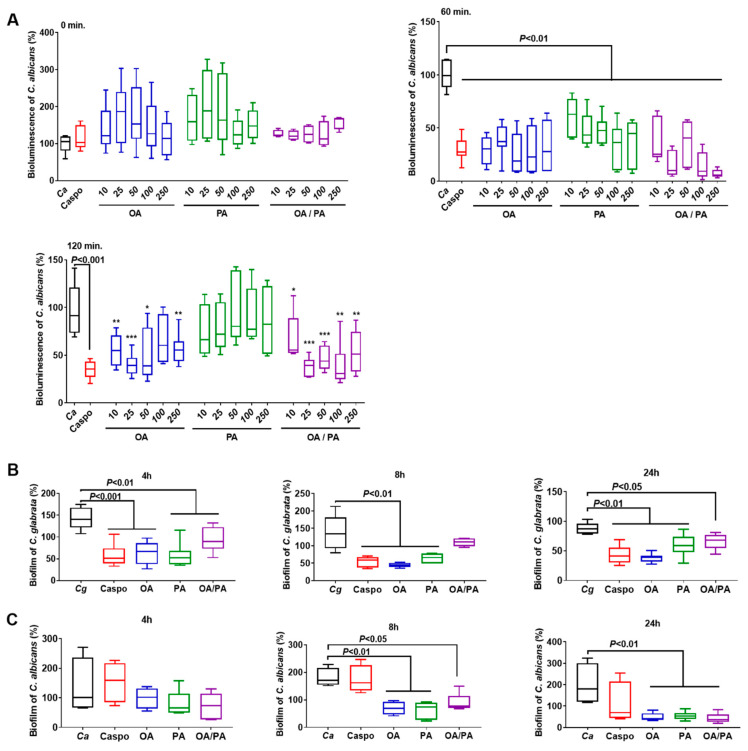
Effect of fatty acids on *Candida* spp. (**A**) Bioluminescence of *C. albicans* treated with OA, PA, or OA/PA at a concentration of 10, 25, 50, 100, or 250 µg/mL after 0, 60, and 120 min. * *p* < 0.05 OA, PA, or OA/PA vs. *C. albicans*; ** *p* < 0.01 OA, PA, or OA/PA vs. *C. albicans*; *** *p* < 0.001 OA, PA, or OA/PA vs. *C. albicans*. (**B**) Biofilm of *C. glabrata* challenged with OA, PA, or OA/PA at a concentration of 25 µg/mL after 4, 8, and 24 h. (**C**) Biofilm of *C. albicans* challenged with OA, PA, or OA/PA at a concentration of 25 µg/mL after 4, 8, and 24 h. *C. glabrata* (*Cg*) and *C. albicans (Ca*): control groups; Caspo: standard control, challenge with caspofungin; OA, PA, OA/PA: *C. glabrata* or *C. albicans* challenged with OA, PA, or FAs (OA/PA), respectively. The results were obtained from three independent experiments.

*B. thetaiotaomicron* and *L. johnsonii* are known to produce chitinase-like and mannosidase-like activities that promote digestion of the *Candida* cell wall, but the question arose whether these two FAs from these bacteria would have a direct effect on *Candida* viability. The effect of these FAs on *Candida* viability was assessed at various concentrations (10, 25, 50, 100, and 250 µg/mL; Figure 6 and Supplementary Materials Figure S3). In parallel, caspofungin, which is an antifungal drug indicated in the treatment of invasive candidiasis, was used as a standard control. At T0, no variation was observed in the bioluminescence of *C. albicans* treated with FAs at different concentrations when compared to untreated *C. albicans* (Figure 6A). However, a significant decrease in the bioluminescence of *C. albicans* was observed after 1 h of coincubation with OA, PA, or FAs at various concentrations. This significant decrease in the bioluminescence of *C. albicans* was only effective for OA or FAs after 2 h of coincubation at a concentration ≥25 µg/mL, indicating that OA exerted a direct antifungal effect against *Candida* when compared to PA. The formation of biofilms, which contain dense yeast cells and hyphae, is an important virulence factor for fungal infection, and correlates strongly with fungal resistance to different antifungal treatments. The effect of FAs on fungal biofilm formation was also determined. Based on the *Candida* viability experiments, FAs at a concentration of 25 µg/mL were used in this biofilm assay. FA treatment significantly decreased biofilm formation by *C. glabrata* and *C. albicans* after 4, 8, or 24 h of incubation (Figure 6B,C), showing that FAs interacted with *Candida* biofilms and affected their formation.

### 3.5. Effect of Fatty Acids on Modulation of the Inflammatory Immune Response and Fungal Overgrowth in a Murine Model of DSS-Induced Colitis

**Figure 7:**
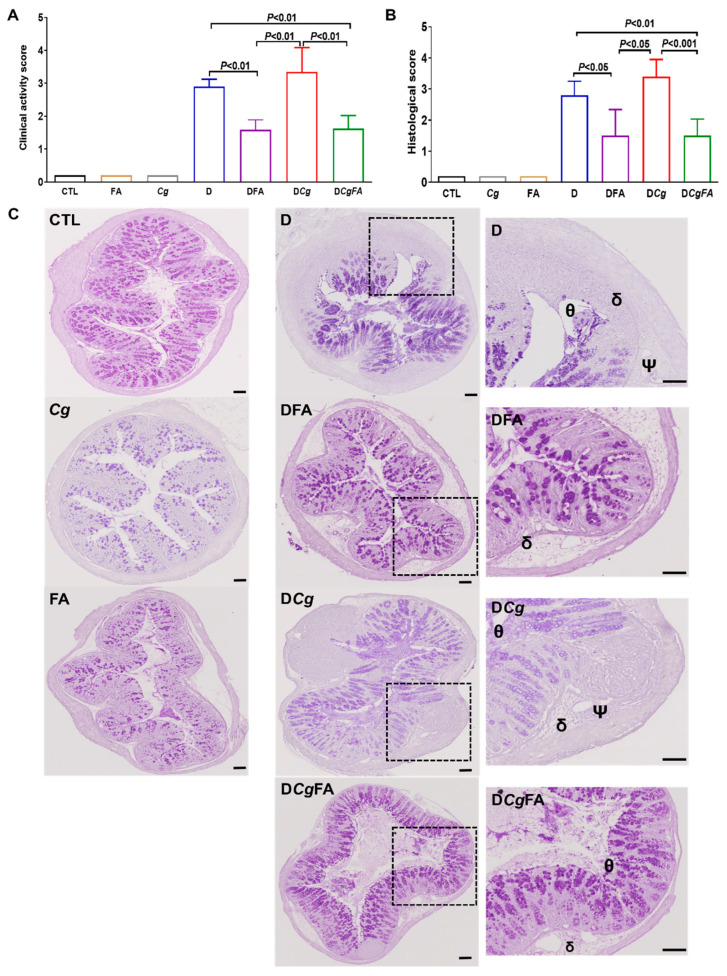
Effect of fatty acids (FAs) on inflammatory parameters in the DSS-induced colitis mouse model. (**A**) Clinical analysis of DSS-induced colitis in mice. Control groups correspond to CTL (water), *Cg* (*C. glabrata*), and FAs (OA and PA). Experimental groups correspond to D (DSS), D*Cg* (DSS and *C. glabrata*), DFA (DSS + OA/PA), and D*Cg*FA (DSS + *C. glabrata* + OA/PA). (**B**) Histologic scores for inflammation. (**C**) Histologic analysis of colon sections from DSS-induced colitis. The symbols Θ, Ψ, δ correspond to epithelial lesions; edema; and leukocyte infiltrate, respectively. Scale bar corresponds to 50 µm.

To assess whether FAs could eliminate *C. glabrata* from the gut during intestinal inflammation, mice were administered an oral inoculum of *C. glabrata* on a day 1, and every mouse was tagged and followed daily in terms of body weight, clinical score for inflammation, and fungal and bacterial load in stool samples. During the experiment, mice received an oral dose of OA and PA at a concentration of 1 mg/kg/mouse daily for 5 days. With regards to inflammatory parameters, control groups corresponding to mice receiving water (CTL), FA alone, and *C. glabrata* (*Cg*) did not show any signs of inflammation in terms of clinical and histologic scores (Figure 7A,B). Furthermore, FA prevented the increase in clinical scores for inflammation for DFA (DSS + FA) and D*Cg*FA (DSS + *Cg* + FA) when compared to groups D (DSS only) and D*Cg* (DSS + *Cg*) (Figure 7A). With regard to the histologic score for inflammation, colon sections from D and D*Cg* mice showed greater leukocyte infiltrate, edema, and epithelial lesions, while FA treatment significantly decreased the signs of inflammation in colons from DFA and D*Cg*FA mice, showing that FA prevented epithelial damage by exerting an anti-inflammatory effect during the development of colitis (Figure 7B,C).

### 3.6. Fungal Load in the Stools and Gut Was Investigated Every 2 Days and in the Digestive Tract on Day 14 (Colon, Cecum, and Stomach)

**Figure 8:**
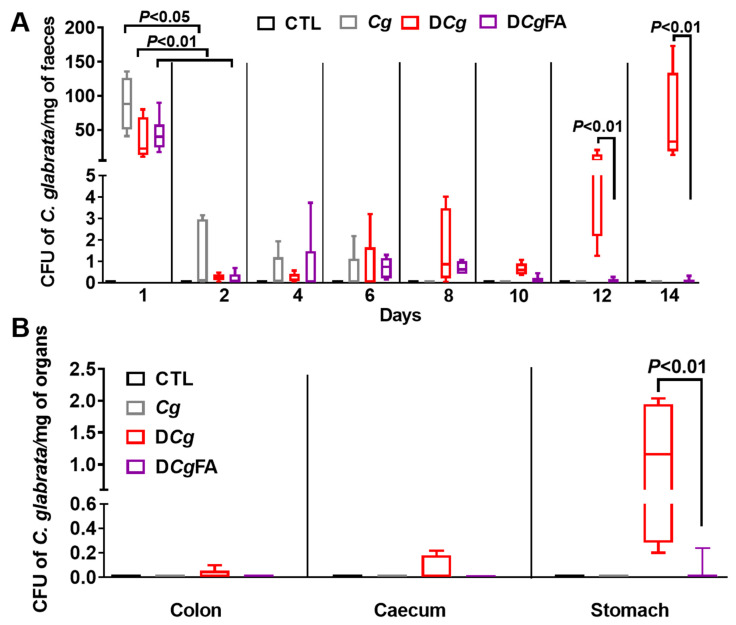
Effect of fatty acids (FAs) on *C. glabrata* elimination from the gut in mice with DSS-induced colitis. (**A**) Determination of the number of *C. glabrata* colonies recovered from stools. (**B**) Number of *C. glabrata* colonies recovered from the stomach, caecum, and colon. The four groups consisted of controls (CTL; water), *C. glabrata* alone (*Cg*), DSS + *C. glabrata* (D*Cg*), and DSS + *C. glabrata* + FAs (D*Cg*FA). Data are the mean ± SD of six mice per group.

No *C. glabrata* was observed in stools or the gut of the CTL group receiving water. For *Cg*, D*Cg*, or D*Cg*FA mice, on day 1, after *C. glabrata* challenge, a significant fungal load in stools was observed in all groups, while a rapid decrease in *C. glabrata* CFU was recorded on day 2 in all groups, indicating that the mice were not natural hosts for *C. glabrata*. This fungal decrease continued until day 6, when elimination of *C. glabrata* was observed in the *Cg* group (control group untreated with DSS) and in mice treated with FAs (D*Cg*FA), while the D*Cg* group had a significant increase in fungal load from day 12, correlating with significant signs of inflammation (Figure 8A). In addition, FA treatment significantly decreased *C. glabrata* in the stomach, indicating that FAs promoted the elimination of *C. glabrata* from the gut (Figure 8B).

**Figure 9:**
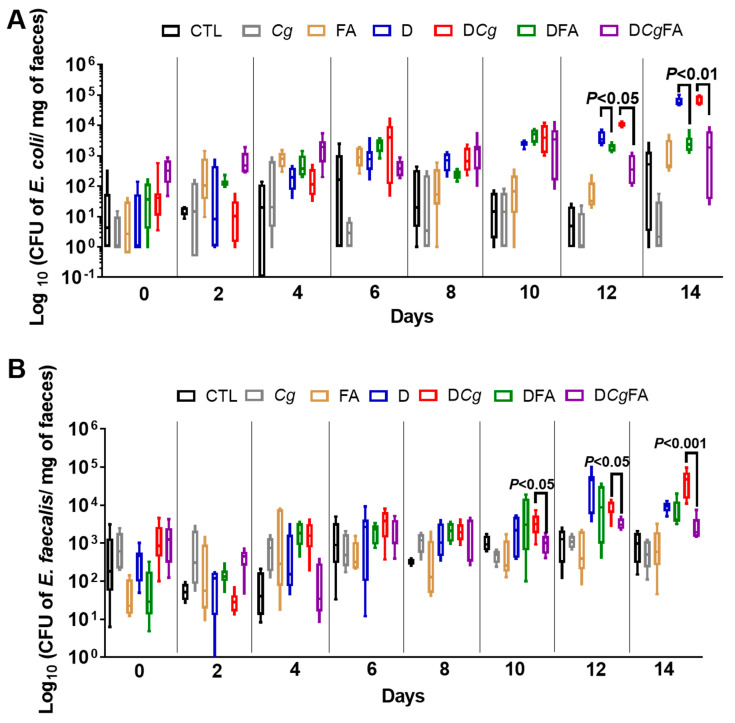
Cultivable *E. faecalis* and *E. coli* after fatty acid (FA) treatment in mice with DSS-induced colitis. (**A**) Number of *E. coli* colony–forming units (CFUs) recovered from stool samples. (**B**) Number of *E. faecalis* CFUs recovered from stool samples. Stool bacteria were isolated from mice on day 0 before *C. glabrata*, DSS challenge, and FA treatment. The seven groups consisted of controls (CTL; water), *C. glabrata* alone (*Cg*), oleic and palmitic acids alone (FA), DSS alone (D), DSS + oleic and palmitic acids (DFA), DSS + *C. glabrata* (D*Cg*), and DSS + *C. glabrata* + oleic and palmitic acids (D*Cg*FA). Data are the mean ± SD of six mice per group.

In terms of the effect of FAs on modulating pathogenic bacteria, in particular *E. coli* and *Enterococcus faecalis*, which are involved in the pathogenesis of IBD, populations of *E. coli* and *E. faecalis* increased significantly during the development of colitis in D and D*Cg* mice when compared to control mice (Figure 9A,B). These bacterial populations significantly decreased in mice treated with FAs (DFA and D*Cg*FA), indicating that FAs affected not only the *Candida* populations, but also the *E. coli* and *E. faecalis* populations.

**Figure 10:**
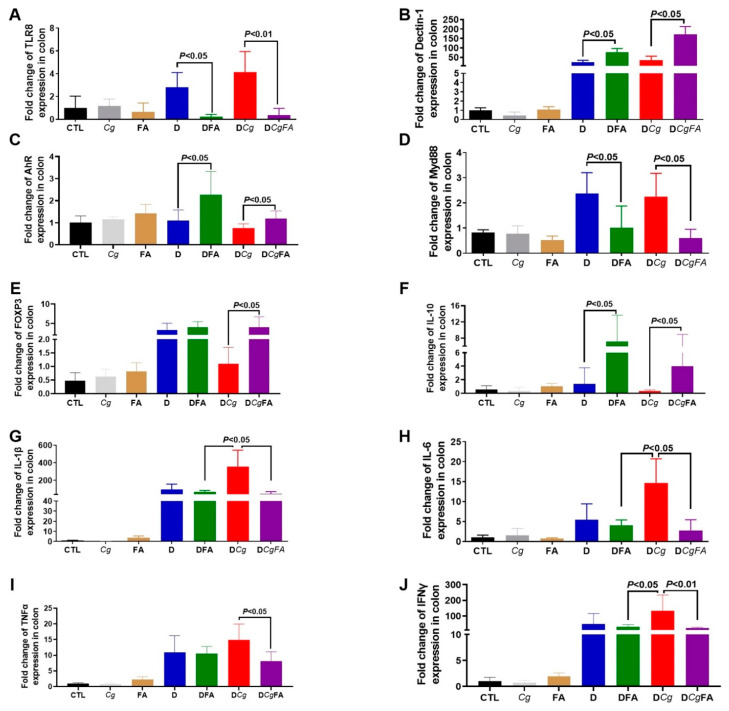
Cytokine and receptor expression after FA treatment. (**A**–**J**) Relative expression levels of TLR8, dectin-1, AhR, Myd88, FOXP3, IL-10, IL-1β, IL-6, TNFα, and IFNγ mRNA in mouse colons. Data are the mean ± SD of six mice per group.

The expression levels of MyD88, FOXP3, and proinflammatory cytokines (IL-1β, IL- 6, TNFα, and IFNγ) were assessed in the colons (Figure 10D,E,G–J). In contrast to FOXP3, expression of MyD88, IL-1β, IL-6, TNFα, and INFγ was significantly lower in the colons of DCgFA mice than in DCg mice, indicating that FAs attenuated the inflammatory response caused by *C. glabrata* overgrowth (Figure 10D,E,G–J). In addition, the expression levels of TLR-8 were significantly decreased, while dectin-1, aryl hydrocarbon receptor (AhR), and IL-10 expression increased in DFA and DCgFA mice (Figure 10A–C,F).

## 4. Discussion

Alteration of the gut microbiota plays a crucial role in many human diseases, including IBD [3,14,30]. Microbial changes in IBD show a decrease in biodiversity of the gut microbiota, in particular strict anaerobic species within the Firmicutes and Actinobacteria phyla [31]. Reduced *Bacteroides* spp. are also observed in IBD, more so in active disease than during remission [32]. The gut microbiota is critical to health and is essential for healthy immune development. Furthermore, some microbial metabolites are key regulators of the host–gut environment, including FAs produced by microbial fermentation [33]. In the murine model of DSS-induced colitis, it has been shown that intestinal inflammation in the gut increased the aerobic bacterial population, in particular *E. coli* and *E. faecalis*, but decreased the population of anaerobic bacteria such as *L. johnsonii* and *B. thetaiotaomicron* [10]. Restoration of these two anaerobic bacteria alleviated the development of colitis in mice, mediated by the modulation of proinflammatory cytokine and TLR expression [11]. Since *L. johnsonii* and *B. thetaiotaomicron* exert a beneficial effect on intestinal inflammation, the present study identified two FAs from these two bacteria during their interaction with colonic epithelial cells. A GC-MS analysis revealed that PA and OA from these two anaerobic bacteria could be detected during their interaction with colonic epithelial cells. PA (16:0) is a saturated fatty acid that is known to modulate the immune system by inducing monocyte activation and stimulating proinflammatory responses in human immune cells [34]. OA (18:1) is an unsaturated fatty acid that exhibits anti-inflammatory properties in humans [35]. Both PA and OA are found in animals, plants, and microorganisms. In the present study, OA alone or OA combined with PA reduced the expression of proinflammatory mediators in Caco-2 cells challenged with DSS. Additionally, OA alone or OA combined with PA improved intestinal epithelial Caco-2 cells through a reduction in macrophage migration during inflammation. PA alone did not exhibit any anti-inflammatory properties on epithelial cells or macrophages when compared to OA. In line with these data, Finucane et al. showed that OA inhibited ATP-induced IL-1β production through an AMPK-dependent mechanism in LPS- and PA-exposed macrophages [36].

OA and PA are mediated through FFAR1, FFAR2, and FFA3 [37,38]. FFARs are critical for metabolic functions and contribute to energy homeostasis and modulation of inflammation [39]. In terms of the role of FFAR in inflammation, FFAR1 signaling induced by a gut microbial metabolite of linoleic acid restored intestinal epithelial barrier impairment [40]. Different studies have demonstrated the role of FFAR2 in modulation of the inflammatory response [38,40]. Activation of FFAR2 inhibited colitis and inflammation, while mice deficient in FFAR2 showed a severe inflammatory response in colitis that was related to an increase in the recruitment of immune cells [41]. In the current study, OA alone or FAs (OA/PA) increased FFAR1, FFAR2, AMPK, and IL-10 expression in macrophages. Impaired AMPK in macrophages has been shown to be associated with greater production of proinflammatory cytokines [42]. Savado et al. reported that the beneficial effect of OA was dependent on the activation of AMPK, a metabolic sensor that modulates inflammation [43]. Additionally, Howe et al. showed that OA and PA differently modulated TLR2-mediated inflammatory responses in macrophages [44]. We observed that OA increased IL-10 expression in macrophages. These observations were consistent with experimental studies, which showed that the anti-inflammatory effects of an OA-enriched diet improved whole-body insulin resistance by reducing the inflammatory response and increasing IL-10 levels in an animal model of diet-induced obesity [45]. The addition of OA alone or FAs to LPS-stimulated macrophages decreased COX-2, TNFα, IL-6, and IL-12 production. In line with these observations, Muller et al. showed that the addition of OA to LPS-stimulated macrophages significantly reduced LPS-induced expression of inducible nitric oxide synthase (iNos), COX-2, and IL-6 expression [46].

In addition to the effect of FAs on modulation of the inflammatory response in macrophages and intestinal epithelial Caco-2 cells, these two FAs have also been shown to exhibit antibacterial and antifungal activity. In the present study, FA treatment significantly decreased *Candida* viability and *Candida* spp. biofilm formation. Muthami et al. showed, using proteomic analysis of *C. albicans*, that OA exerted stress conditions such as heat stress and targeted the proteins implicated in glucose metabolism, ergosterol biosynthesis, lipase production, iron homeostasis, and amino acid biosynthesis [47]. Like OA, PA inhibited the virulence factors of *Candida* spp. such as ergosterol biosynthesis, enzymatic activity, and mature biofilm formation at various time points [48].

Given that OA and PA were detected during the interaction of *L. johnsonii* and *B. thetaiotaomicron* with colonic epithelial cells, and the combination of OA and PA had anti-inflammatory and antifungal properties in vitro, these FAs were evaluated in the murine model of DSS-induced colitis. FA prevented the increase in clinical and histologic scores for inflammation and allowed the elimination of *C. glabrata* from the gut. This observation was supported by a reduction in the *E. coli* and *E. faecalis* populations in mice treated with FAs, indicating that these FAs were not only able to decrease *C. albicans* colonization, but also attenuated intestinal inflammation. Reddy at al. showed that the antioxidant activity of OA inhibited mucosal damage, as evidenced by lower crypt distortion, edema, and abundant goblet cells in the mucosa [49]. In this animal model, increased expression of IL-1β and IL-6 was associated with the development of inflammation and *C. glabrata* overgrowth in mice, while FA attenuated the expression of these cytokines. Ben-Dror et al. showed that the combination of OA and PA significantly alleviated cellular stress and inflammation marker levels [50]. Additionally, the expression of TLR8 and MyD88 decreased in mice treated with FAs. This reduction was correlated with the reduction in *C. glabrata*, *E. coli*, and *E. faecalis* populations in mice treated with FAs, showing that the effect of FAs on pathogens modulated the inflammatory response mediated via TLR/MyD88. In contrast, FA treatment increased dectin-1 expression in the colons of D*Cg* mice. This observation was in line with a previous study showing that dectin-1 expression was increased during the elimination of *C. glabrata* in mice with DSS-induced colitis [10]. Expression of AhR, which is a receptor for aromatic hydrocarbons, was suppressed in the intestines of patients with IBD [51,52]. AhR −/− mice exhibited severe inflammatory parameters in DSS-induced colitis and produced high levels of proinflammatory cytokines, suggesting that the function of AhR in the intestine is protective [53]. In the present study, we observed that FAs increased the expression of AhR in mice treated with DSS or DSS+*C. glabrata,* and this increase in AhR expression was correlated with the increase in IL-6, TNFα, and IFNγ. Lamas et al. showed that the administration of three *Lactobacillus* strains to mice promoted the activation of AhR by rescuing impaired IL-22 production and allowed the attenuation of intestinal inflammation [54].

In conclusion, OA and PA were detected during the interaction of *L. johnsonii* and *B. thetaiotaomicron* with colonic epithelial cells. OA alone or FAs reduced the expression of proinflammatory mediators in Caco-2 cells challenged with DSS. OA alone or FAs increased FFAR1, FFAR2, AMPK, and IL-10 expression in macrophages. Additionally, OA alone or FAs decreased COX-2, TNFα, IL-6, and IL-12 expression in LPS-stimulated macrophages. In the DSS-induced colitis model, oral administration of FAs prevented the increase in clinical and histologic scores for inflammation, reduced the *E. coli* and *E. faecalis* populations, and eliminated *C. glabrata* from the gut. Overall, these findings provided evidence that FAs have anti-inflammatory and antifungal properties. These two FAs decreased the inflammatory response in macrophages mediated via FFAR1, FFAR2, FFAR3, and AMPK; attenuated the development of colitis; and were involved in the elimination of *C. glabrata* from the gut. This study emphasized the importance of adding unsaturated FAs, in particular OA, to the human diet to promote the attenuation of intestinal inflammation and fungal elimination from the gut. In addition, the current work offers new perspectives for exploring the effect of polysaturated and trans isomers of both OA and PA on fungal overgrowth, as well as how these FAs can modulate the microbiota biodiversity and intestinal inflammation.

## Data Availability

The data that support the findings of this study are available on https://entrepot.recherche.data.gouv.fr/dataverse/olipalm.

## Supplementary Materials

The following supporting information can be downloaded at: https://www.mdpi.com/article/10.3390/microorganisms10091803/s1, Figure S1. Expression of pro-inflammatory mediators and receptors in macrophages treated with fatty acids and challenged with LPS; Figure S2. Protein levels of TNFα, IL-6 and IL-1β in macrophages treated with fatty acids and exposed to LPS; Figure S3. Effect of fatty acids on viability of *C. glabrata* by culture plate assay; Table S1. Quantitative real-time RT-PCR primer sequences for human genes used in the study; Table S2. Mouse primers used for PCR analysis, related to experimental procedures.
