# Supplementary material for: Oleic Acid and Palmitic Acid from Bacteroides thetaiotaomicron and Lactobacillus johnsonii Exhibit Anti-Inflammatory and Antifungal Properties
Source: Microorganisms. 2022 Sep 8;10(9):1803. doi: 10.3390/microorganisms10091803 (PMC9504516; doi:10.3390/microorganisms10091803)
Supplement: Supplementary file 1 [file microorganisms-10-01803-s001.zip › microorganisms-1639011-supplementary.pdf]

microorganisms

MDPI

Supplementary materials

We checked for additional pro-inflammatory cytokines using the qRT-PCR approach. OA did not have any effect on the expression of IFN**γ**, IL8, IL-17, or IL-18 while PA increased the expression of IFN**γ** and IL-17 in LPS-stimulated macrophages (Figure 1). For the protein level approach, the combination OA with PA reduced the expression of TNFα and IL-6 (Figure 2).

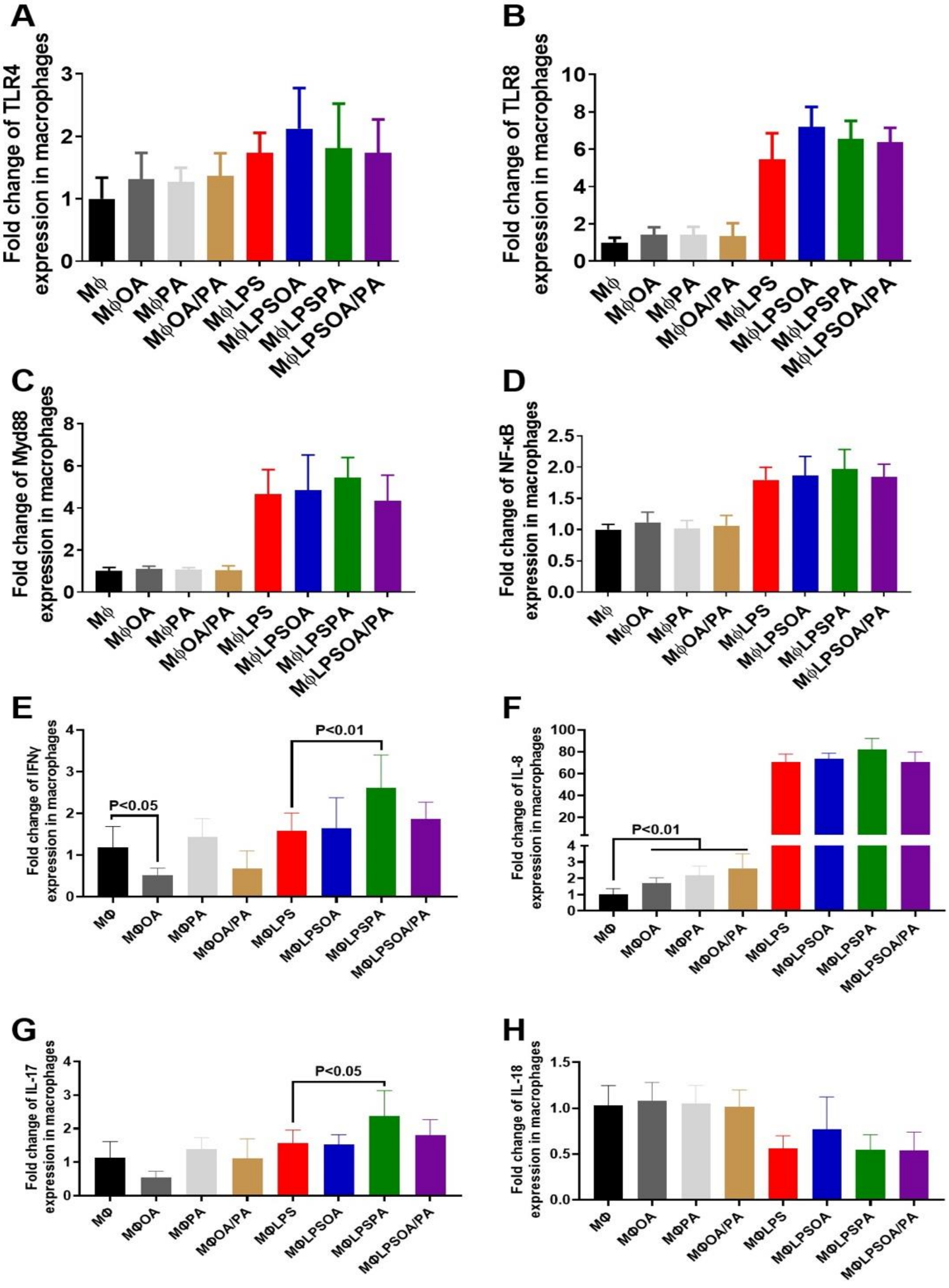

Figure S1. Expression of pro-inflammatory mediators and receptors in macrophages treated with fatty acids and challenged with LPS. (A-H) Relative expression levels of TLR4, TLR8, Myd88, NF-

κB, IFNγ, IL-8, IL-17 and IL-18 mRNA, respectively in macrophages. MΦ represents a control group (macrophages alone); MΦOA, MΦPA and MΦOA/PA correspond to macrophages treated with oleic acid, palmitic acid or with these two fatty acids, respectively. MΦLPS represents a positive control (macrophages exposed to lipopolysaccharide). MΦLPSOA, MΦLPSPA and MΦLPSOA/PA correspond to macrophages challenged with LPS and treated with oleic acid, palmitic acid or with these two fatty acids, respectively. The results were obtained from three independent experiments.

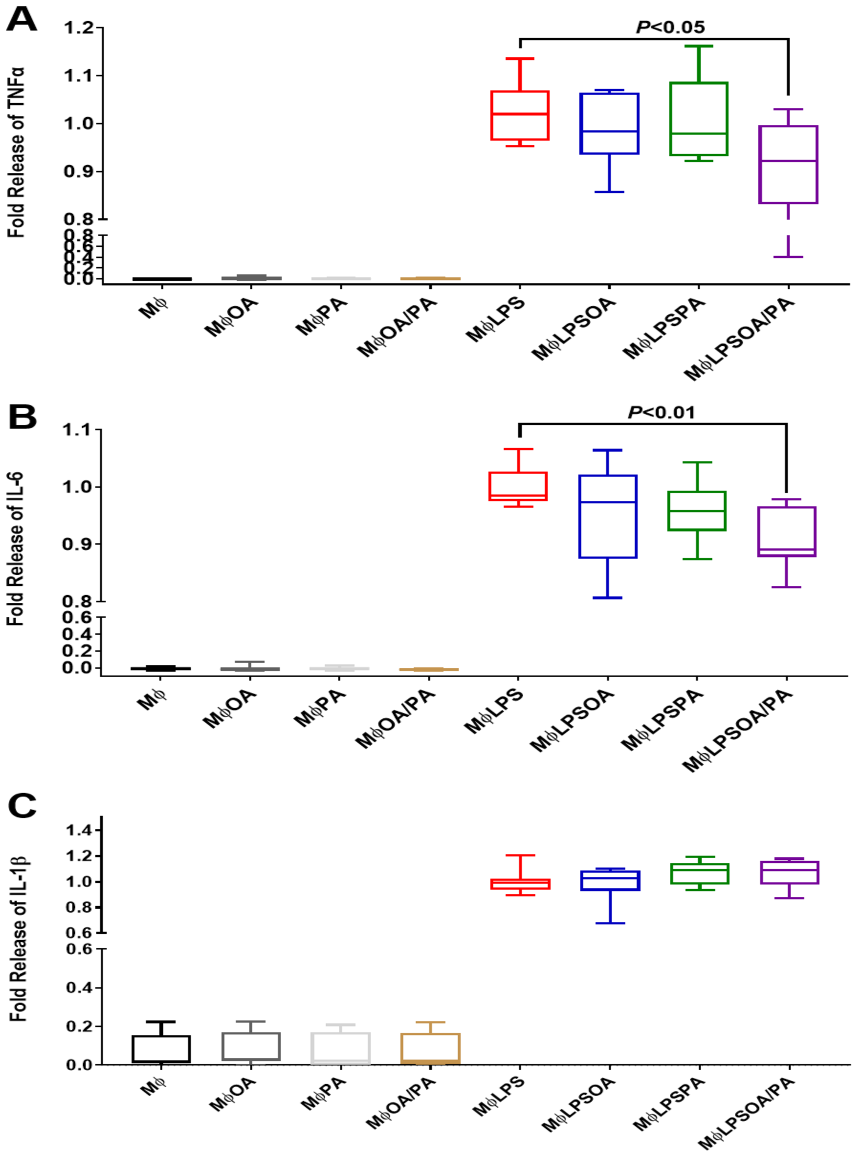

Figure S2. Protein levels of TNFα, IL-6 and IL-1β in macrophages treated with fatty acids and exposed to LPS. (A) Fold release of TNFα in macrophages. (B) Fold release of IL-6 in macrophages. (C) Fold release of IL-1β. MΦ represents a control group (macrophages alone); MΦOA, MΦPA and MΦOA/PA correspond to macrophages treated with oleic acid, palmitic acid or with these two fatty acids, respectively. MΦLPS represents a positive control (macrophages exposed to lipopolysaccharide). MΦLPSOA, MΦLPSPA and MΦLPSOA/PA correspond to macrophages challenged with LPS and treated with oleic acid, palmitic acid or with these two fatty acids, respectively. The results were obtained from three independent experiments.

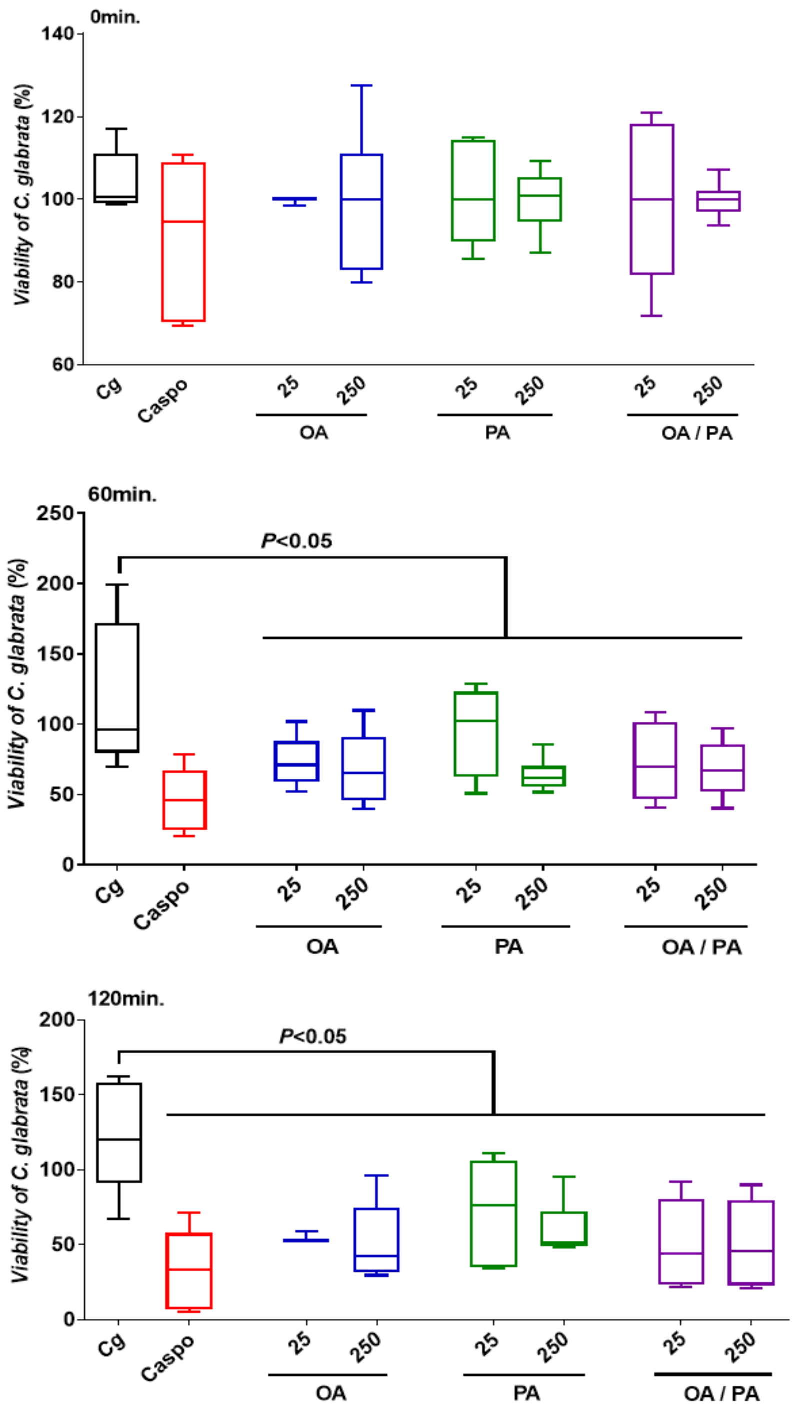

Figure S3. Effect of fatty acids on viability of *C. glabrata* by culture plate assay. *C. glabrata* challenged with OA, PA, OA/PA at a concentration of 25 and 250 µg/mL after 0, 60 and 120 min. *Cg* represents a control. Caspo represents a standard control (*C. glabrata* challenged with caspofungin). OA, PA and OA/PA correspond to *C. glabrata* challenged with oleic acid, palmitic acid or these two FA, respectively. The results were obtained from two independent experiments.

Table S1. Quantitative real-time RT-PCR primer sequences for human genes used in the study.

Gene	Primer sequence (5'-3')	
GAPDH	Forward	GAC-ACC-CAC-TCC-TCC-ACC-TTT
	Reverse	TTG-CTG-TAG-CCA-AAT-TCG-TTG-T
TLR2	Forward	GCC-AAA-GCT-TTG-ATT-GAT-TGG
	Reverse	TTG-AAG-TTC-AGC-TCC-TG
TLR4	Forward	CAG-GGC-TTT-TCT-GAG-TCG-TC
	Reverse	TGA-GCA-GTC-GTG-CTG-GTA-TC
TLR8	Forward	TCC-TTC-AGT-CGT-CAA-TGC-TG
	Reverse	CGT-TTG-GGG-AAC-TTC-CTG-TA
FFAR1	Forward	CCG-GCA-TTC-TAG-CTC-AGG-AG
	Reverse	CAC-TCT-GCC-CCT-TCC-GAA-TT
FFAR2	Forward	TGC-CTT-TTT-GAT-GTG-CTC-TG
	Reverse	TTG-CTT-TCC-CCT-GTA-TGA-GG
FFAR3	Forward	TCT-CAG-CAC-CCT-GAA-CTC-CT
	Reverse	TTC-TGC-TCC-TTC-AGC-TCC-AT
AMPKα	Forward	AAC-AAG-TTG-TGG-CTC-ACC-CA
	Reverse	AGA-ATC-AGG-TGG-GCT-TGT-CG
COX-2	Forward	TGA-AAC-CCA-CTC-CAA-ACA-CA
	Reverse	GAG-AAG-GCT-TCC-CAG-CTT-TT
NF-κB	Forward	AGT-GAA-CCG-AAA-CTC-TGG-CA
	Reverse	CCT-GGT-CCC-GTG-AAA-TAC-A
Myd88	Forward	GGA-ATG-TGA-CTT-CCA-GAC-CAA
	Reverse	GAT-GGG-GAT-CAG-TCG-CTT-C
AhR	Forward	GCA-CCG-ATG-GGA-AAT-GAT-ACT-ATC
	Reverse	GGT-GAC-CTC-CAG-CAA-ATG-AGT-T
TNFα	Forward	ATC-AAT-CGG-CCC-GAC-TAT-CTC
	Reverse	ACA-GGG-CAA-TGA-TCC-CAA-AGT
IFNγ	Forward	TCC-CAT-GGG-TTG-TGT-GTT-TA
	Reverse	AAG-CAC-CAG-GCA-TGA-AAT-CT
IL-1β	Forward	GAT-GCA-CCT-GTA-CGA-TCA-CT
	Reverse	GAC-ATG-GAG-AAC-ACC-ACT-TG
IL-6	Forward	AGT-GAG-GAA-CAA-GCC-AGA-GC
	Reverse	GTC-AGG-GGT-GGT-TAT-TGC-AT
IL-8	Forward	AAA-TCA-GGA-AGG-CTG-CCA-AGA
	Reverse	AAG-GAA-CCA-TCT-CAC-TGT-GTG-TAA-AC
IL-10	Forward	ACT-TTA-AGG-GTT-ACC-TGG-GTT-GC
	Reverse	TCA-CAT-GCG-CCT-TGA-TGT-CTG
IL-12	Forward	CCT-GAC-CAT-CCA-AGT-CAA-AGA-GT
	Reverse	AGG-AGC-GAA-TGG-CTT-AGA-ACC-T
IL-17	Forward	TCC-CAC-GAA-ATC-CAG-GAT-GC
	Reverse	GGA-TGT-TCA-GGT-TGA-CCA-TCA-C
IL-18	Forward	AGT-CAG-CAA-GGA-ATT-GTC-TCC
	Reverse	GAA-GCG-ATC-TGG-AAG-GTC-TG
CCL2	Forward	CCC-CAG-TCA-CCT-GCT-GTT-AT
	Reverse	TGG-AAT-CCT-GAA-CCC-ACT-TC
CCL5	Forward	CGC-TGT-CAT-CCT-CAT-TGC-TA
	Reverse	CCA-GAC-TTG-CTG-TCC-CTC-TC

Table S2. Mouse primers used for PCR analysis, related to experimental procedures.

Gene	Primer sequence (5'-3')	
Polr2a	Forward	CCC-ACA-ACC-AGC-TAT-CCT-CAA
	Reverse	GGT-GCT-GTG-GGT-ACG-GAT-ACA
TLR8	Forward	GGC-ACA-ACT-CCC-TTG-TGA-TT
	Reverse	CAT-TTG-GGT-GCT-GTT-GTT-TG
Dectin-1	Forward	GGG-CTC-TCA-AGA-ACA-ATG-GA
	Reverse	AGC-ACA-CGA-TCC-TTT-CTC-TG
Myd88	Forward	ACT-GGC-CTG-AGC-AAC-TAG-GA
	Reverse	CGT-GCC-ACT-ACC-TGT-AGC-AA
Ahr	Forward	TGC-TGG-TGT-CTG-CCA-TTG-TC
	Reverse	ACA-GAT-CCA-GGA-CCA-CAT-CCT-T
FOXP3	Forward	CAA-CAT-GGA-CTA-CTT-CAA-GTA-CCA-CAA-TA
	Reverse	GAT-GGC-CCA-TCG-GAT-AAG-G
TNFα	Forward	CCA-CCA-CGC-TCT-TCT-GTC-TA
	Reverse	GAG-GCC-ATT-TGG-GAA-CTT-CT
IL-1β	Forward	CAG-CAA-CAG-CAA-GGC-GAA-A
	Reverse	CTG-GAC-CTG-TGG-GTT-GTT-GAC
IFNγ	Forward	CAG-CAA-CAG-CAA-GGC-GAA-A
	Reverse	CTG-GAC-CTG-TGG-GTT-GTT-GAC
IL-6	Forward	TAC-ACA-TGT-TCT-CTG-GGA-AAT-CGT
	Reverse	AAG-TGC-ATC-ATC-GTT-GTT-CAT-ACA
IL-10	Forward	CAG-TAC-AGC-CGG-GAA-GAC-AAT-AA
	Reverse	CCG-CAG-CTC-TAG-GAG-CAT-GT
